# Simultaneous Detection of Both RNA and DNA Viruses Infecting Dry Bean and Occurrence of Mixed Infections by BGYMV, BCMV and BCMNV in the Central-West Region of Mexico

**DOI:** 10.3390/v9040063

**Published:** 2017-03-30

**Authors:** Elizabeth Chiquito-Almanza, Jorge A. Acosta-Gallegos, Nadia C. García-Álvarez, Eduardo R. Garrido-Ramírez, Victor Montero-Tavera, Lorenzo Guevara-Olvera, José L. Anaya-López

**Affiliations:** 1 Instituto Tecnológico de Celaya, Departamento de Ingeniería Bioquímica, Celaya, Guanajuato, CP 38010, Mexico; ely_sayra@hotmail.com (E.C.-A.); lorenzogo@yahoo.com (L.G.-O.); 2 INIFAP-Campo Experimental Bajío, Celaya, Guanajuato, CP 38010, Mexico; acosta.jorge@inifap.gob.mx (J.A.A.-G.); montero.victor@inifap.gob.mx (V.M.-T.); 3 INIFAP-Campo Experimental Santiago Ixcuintla, Santiago Ixcuintla, Nayarit, CP 63300, Mexico; garcia.nadia@inifap.gob.mx; 4 INIFAP-Campo Experimental Centro de Chiapas, Ocozocoautla, Chiapas, CP 29140, Mexico; garrido.eduardo@inifap.gob.mx

**Keywords:** *Phaseolus vulgaris*, virus detection, multiplex RT-PCR

## Abstract

A multiplex reverse transcription polymerase chain reaction (RT-PCR) assay was developed to simultaneously detect bean common mosaic virus (BCMV), bean common mosaic necrotic virus (BCMNV), and bean golden yellow mosaic virus (BGYMV) from common bean leaves dried with silica gel using a single total nucleic acid extraction cetyl trimethyl ammonium bromide (CTAB) method. A mixture of five specific primers was used to amplify three distinct fragments corresponding to 272 bp from the *AC1* gene of BGYMV as well as 469 bp and 746 bp from the *CP* gene of BCMV and BCMNV, respectively. The three viruses were detected in a single plant or in a bulk of five plants. The multiplex RT-PCR was successfully applied to detect these three viruses from 187 field samples collected from 23 municipalities from the states of Guanajuato, Nayarit and Jalisco, Mexico. Rates of single infections were 14/187 (7.5%), 41/187 (21.9%), and 35/187 (18.7%), for BGYMV, BCMV, and BCMNV, respectively; 29/187 (15.5%) samples were co-infected with two of these viruses and 10/187 (5.3%) with the three viruses. This multiplex RT-PCR assay is a simple, rapid, sensitive, and cost-effective method for detecting these viruses in the common bean and can be used for routine molecular diagnosis and epidemiological studies.

## 1. Introduction

Virus diseases are among the major biotic constraints to legume production, particularly in the tropics and subtropics [1,2]. Common bean (*Phaseolus vulgaris* L.) is likely the most susceptible plant species in the Leguminosae to virus infection [3] and one of the most widely cultivated legumes in the world. In 2013, over 29 million hectares of tropical and temperate agricultural land in America, Europe, Africa and Asia were used for common bean production [4]. Among the viruses infecting beans, the potyviruses bean common mosaic virus (BCMV) and bean common mosaic necrosis virus (BCMNV) are most worldwide spread, and together with the geminiviruses bean golden mosaic virus (BGMV) and bean golden yellow mosaic virus (BGYMV) are considered one of the major production constraints of common bean in Latin America, the Caribbean, and East, West and Southern Africa [5,6]. BCMV and BCMNV are seed-borne, with a percentage of seed transmission as high as 50% [7] and hence are readily spread within and between countries [8,9]. BCMV can remain viable in bean seeds for more than 30 years [9], and under field conditions the infected seedlings originated from BCMV- or BCMNV-infected seeds act as primary foci, subsequently the virus spreads through several aphid species in a non-persistent manner [10]. Yield losses of 53–83% in beans have been attributed to BCMV [11], and even symptomless BCMV infections can induce yield losses higher than 50% in susceptible common bean genotypes [3].

BGYMV can cause significant yield losses in susceptible bean cultivars, as this virus infects the common bean in southern Mexico, Central America and the Caribbean [12], it is not seed-transmitted [12,13] but is an important bean production constraint in countries such as Guatemala and El Salvador, particularly in years with a long dry season during the drier months of the year (November–March) and an endemic but sporadic production problem in Costa Rica and Panama in years with an average rainfall above 1500 mm [12]. Disease incidence and the extent of losses vary depending on populations of the whitefly vector (*Bemisia tabaci* (Genn.)), susceptibility of the bean cultivar and environmental conditions, mainly rainfall, which affects *B. tabaci* populations and cultural practices [12].

The accurate detection and identification of the virus affecting the crop is necessary for the implementation of suitable disease management strategies [11] and for the development of new resistant cultivars. During the fall-winter cycle of 2013/14, single and mixed infections of BCMV, BCMNV and a begomovirus, subsequently identified through sequencing as BGYMV, were detected in samples collected from irrigated and rainfed commercial fields in Nayarit, Mexico through reverse transcription polymerase chain reaction (RT-PCR) and PCR with primers for the genus *Begomovirus* and specific primers for seven seed-borne viruses [14].

Specific polyclonal antisera, monoclonal antibodies, RT-PCR, PCR and recombinase polymerase amplification (RPA) have been utilized to detect, differentiate, and individually characterize BCMV, BCMNV or BGYMV in each specific reaction [9,15,16], but these techniques are time-consuming, laborious, and costly when large numbers of samples are tested for mixed infections. These limitations can be overcome through reliable field sampling and a multiplex viral detection method, such as multiplex RT-PCR, which can simultaneously and specifically amplify more than one target sequence of RNA and DNA viruses using total nucleic acids as a template, allowing investigation of the epidemiology of common bean viruses easier. To date, multiplex RT-PCR for the simultaneous detection of RNA and DNA plant viruses through RT-PCR using total nucleic acids as a template has been used to diagnose infections in black pepper (*Piper nigrum*) [17], strawberry (*Fragaria* spp.) [18] and cassava (*Manihot esculenta* Crantz) [19], and this method significantly reduces the time and cost of detection compared to individual virus detection. However, this method has not been applied to the simultaneous detection of BCMV, BCMNV and BGYMV, three of the most important viruses affecting the production of the common bean. In the present work, we describe the development and evaluation of a multiplex RT-PCR assay for the simultaneous detection and differentiation of BCMV, BCMNV and BGYMV in single and mixed infections of the common bean.

## 2. Materials and Methods

### 2.1. BCMV, BCMNV and BGYMV Virus Strains

The BCMV NL-4 and BCMNV NL-3 strains (kindly provided by the Virology Research Unit of CINVESTAV-Irapuato, Mexico), preserved in the seeds of cultivars Red Mexican-34 and Michelite-62, respectively, were used as positive controls for developing RT-PCR assays. Common bean seeds infected with each virus were planted in a containment greenhouse, and leaf tissues of plants that developed common mosaic symptoms were used for total nucleic acid extraction. The BGYMV-MX strain, GenBank Accession number AF173555 (component A) and AF173556 (component B) [20], were used as a positive control for developing the PCR assays. Ten-day-old bean seedlings were agroinoculated with a mixture of BGYMV-MX component A and BGYMV-MX component B *Agrobacterium* cell suspensions through needle puncture-injection of the first internode. Leaf tissues from plants with golden mosaic symptoms were used for total nucleic acid extraction.

### 2.2. Simultaneous Extraction of Both Total RNA and Total DNA

A single nucleic acid extraction protocol for both DNA and RNA virus extractions was used following the cetyl trimethyl ammonium bromide (CTAB) method according to Abarshi et al. [21]. In summary, ~30 mg of common bean leaf tissue dried with silica gel was placed in a 1.5 mL Eppendorf tube, frozen with liquid nitrogen and ground using a frozen polypropylene pestle. Subsequently, the powder was mixed with 750 µL of CTAB buffer (Affymetrix USB, Santa Clara, CA, USA). The integrity of the extracted nucleic acids was determined by analyzing 1 µL of the extract using 1% TAE (Tris base, Invitrogen, Carlsbad, CA, USA; acetic acid, J.T.Baker, Center Valley, PA, USA; and ethylenediaminetetraacetic acid, (EDTA), J.T. Baker, Center Valley, PA, USA), agarose gel electrophoresis (Invitrogen), stained with 1X GelRed (Biotium Inc., Fremont, CA, USA) and visualized under UV light using a Gel Logic 212 Pro System (Carestream Molecular Imaging, New Haven, CT, USA), and the quantity and quality were determined through spectrophotometry at 260 nm and 280 nm wavelengths using a Nanodrop 8000™ (Thermo Fisher Scientific, Waltham, MA, USA).

### 2.3. Reverse Transcription

For all the samples, reverse transcription (RT) was conducted using 1 µg of total nucleic acids extracted from common bean plants infected with each virus strain and a SuperScript™ III First-Strand synthesis kit (Invitrogen, Carlsbad, CA, USA) in a final volume of 20 µL according to the manufacturer’s instructions. The RT reactions were stored at −20 °C for uniplex, duplex and multiplex PCR reactions. Since cDNA was synthesized from the total RNA contained in the total nucleic acids extraction, the DNA components of BGYMV remained in the reaction mix of the synthesis of the samples infected with this virus; thus, these mixtures cDNA/DNA were used as a template for BGYMV PCR.

### 2.4. Design of New Primers for BGYMV Detection

The size of the amplicons obtained with primer pair for BGYMV detection (707 base pair; bp) [22] was similar to the PCR products obtained with the primer pair used for BCMNV detection (746 bp) [14]; thus, two pairs of BGYMV component A-specific primers were designed (Table 1) to improve the multiplex detection of BCMV, BCMNV and BGYMV. The genomic sequences of the BGYMV component A were downloaded from the National Center for Biotechnology Information (NCBI) GenBank (Accession numbers: AF173555.1, AJ544531.1, D00200.1, DQ119824.1, L01635.1, M10070.1, M91604.1 and NC_001439.1), aligned with MegAlign V7.0.0. (DNASTAR, Madison, WI, USA), and used to design primers directed to the conserved region of the *AC1* gene for Replication-associated protein (Rep) using PrimerSelect V7.0.0. (DNASTAR, Madison, WI, USA) and Oligoanalyzer [23]. The specificity of the designed BGYMV primers was confirmed in silico using BLASTn V2.3.0 [24]; the amplified PCR products were sequenced, and their identities were compared with the GenBank sequences using BLASTn.

### 2.5. Specificity of the Primers Designed for BGYMV Detection

To validate the specificity of the sets of primers designed in the present study and those of Potter et al. [22] for BGYMV detection (Table 1), PCR analysis were performed using, as positive controls the cDNA from the leaf tissue of common bean infected with BGYMV-MX, the component A of the infectious clone of BGYMV-MX [20] and cDNA from common bean samples with symptoms of bean yellow golden mosaic virus collected in Nayarit, Mexico. The infection of these samples with BGYMV was confirmed after sequencing the fragments amplified using the degenerate primers according to Rojas et al. [25] (Table 1). As negative controls we used the component B of the infectious clone of BGYMV-MX [20] and the of components A and B of each one of the infectious clones of the geminiviruses pepper golden mosaic virus (PepGMV), previously referred to as texas pepper geminivirus (TPV) [26], and pepper huasteco yellow vein virus (PHYVV) [27].

All PCR amplifications were performed in 15 µL reactions containing 1 µL (~10 ng) of the corresponding template, 1X PCR buffer, and 1 U of *Taq* DNA polymerase recombinant (Invitrogen). The PCRs were conducted with the primer sets according to Rojas [25] and Potter [22] (Table 1) containing 2.5 mM MgCl_2_, 0.2 µM of each specific primer and 0.2 mM each of dNTPs. The PCR programme included one denaturation cycle at 95 °C for 3 min, followed by 30 cycles at 94 °C for 45 s, 55 °C for 2 min and 72 °C for 2 min, with a final extension step at 72 °C for 3 min. The PCRs with primer sets designed in the present study for BGYMV detection included 1.5 mM MgCl_2,_ 0.1 µM of each specific primer and 0.3 mM each of dNTPs. The PCR programme included one denaturation cycle at 94 °C for 3 min, followed by 30 cycles at 94 °C for 45 s, 64 °C for 30 s and 72 °C for 1 min, with a final extension step at 72 °C for 7 min.

For all PCR reactions, 1 µL of nuclease free water and 1 µL of the cDNA from a healthy plant were included as a non-template control (NTC) and a negative control, respectively. All PCRs were conducted using a T-100 Thermal Cycler (Applied Biosystems, Foster City, CA, USA), and aliquots of 1 µL of the PCR were analysed as previously described in Section 2.2 to confirm that the amplicons had the expected size (Table 1).

### 2.6. Optimization of Uniplex, Duplex and Multiplex PCR for BCMV, BCMNV and BGYMV Detection

The cDNA from foliar tissues of common bean infected with BCMV-NL4, BCMV-NL3 and BGYMV-MX strains were used as positive controls. Seven different templates were used for each uniplex, duplex and multiplex PCR: three single-virus templates comprised 1 µL of each cDNA of the positive controls; three double-virus templates comprised 1 µL of an equimolar mixture of two positive controls, and one triple-virus template comprised 1 µL of an equimolar mixture of the three positive controls. The uniplex, duplex and multiplex PCRs were performed using the combination of the primers specific for each virus; for the detection of BCMV and BCMNV, we used the primer sets D1BCMV & RU and D1BCMNV & RU [14], respectively, and for BGYMV we used the specific primer pair DMexBGYMV & RMexBGYMV (Table 1). The conditions and PCR programme were the same as described for the BGYMV detection with the primers designed in this research.

### 2.7. Composite Sampling

To determine the suitability of a multiplex protocol for detecting BCMV, BCMNV and BGYMV in composite samples, 1 µL of 1:1, 1:2, 1:3, 1:4, 1:5 and 1:6 dilutions of a triple-virus infected sample, diluted in the cDNA from healthy plants, was used as the template in multiplex PCR reactions. Aliquots of 1 µL of the PCR were resolved using 1% TAE agarose gel electrophoresis to confirm the size of the amplicons.

### 2.8. Multiplex Detection of BCMV, BCMNV and BGYMV in Samples Collected in Crop Fields

Foliar tissue from common bean plants displaying virus-like symptoms were collected in irrigated and rainfed commercial bean fields from Guanajuato and Jalisco, Mexico during the spring–summer growing season of 2014 and from Nayarit, Mexico during the fall-winter growing seasons of 2013/2014 and 2014/2015 (Table S1). From each plant, three or four young trifoliates were collected, preferably from the leaves where the central leaflet measured from 0.6 to 2 cm in length, and the samples were immediately placed between two lens papers (CHE Scientific, Kwai Chung, Hong Kong), transferred to a plastic Zip-lock bag (10 × 15 cm) with 25 g of silica gel orange indicator (Golden Bell, Cuauhtémoc, D.F, México), and stored in a hermetic container. After two days, the hydrated silica gel was replaced with 10 g of new silica. The plastic Zip-lock bags were stored in a hermetic plastic container at −80 °C until subsequent use for nucleic acids extraction. A total of 187 samples (76 samples from Guanajuato, 39 samples from Jalisco, and 73 samples from Nayarit) were used to detect BCMV, BCMNV and BGYMV. Samples that did not amplify any fragment were tested using PCR for the presence of reference gene 26S ribosomal RNA (rRNA) according to Ruiz-Nieto et al. [28].

## 3. Results

### 3.1. Specificity of the Primers Designed for BGYMV Detection

**Figure 1:**
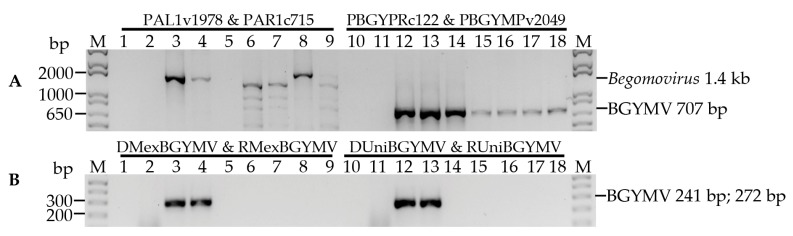
Specificity of four primer pairs for the detection of bean golden yellow mosaic virus (BGYMV) using polymerase chain reaction (PCR). The primer set used in each case is indicated above the lanes (**A**) Primer pairs reported for detection of the genus *Begomovirus* and for specific detection of BGYMV; (**B**) Primer pairs designed in the present study specifically for BGYMV. Lanes 1 and 10: non template control; lanes 2 and 11: negative control (cDNA from healthy plant); lanes 3 and 12: positive control (cDNA from common bean infected with BGYMV-MX strain); lanes 4 and 13: plasmid DNA of BGYMV-MX DNA A component; lanes 5 and 14: plasmid DNA of BGYMV-MX DNA B component; lanes 6 and 15: plasmid DNA of pepper huasteco yellow vein virus (PHYVV) DNA A component; lanes 7 and 16: plasmid DNA of PHYVV DNA B component; lanes 8 and 17: plasmid DNA of pepper golden mosaic virus (PepGMV) DNA A component; lanes 9 and 18: plasmid DNA of PepGMV DNA B component. M: Molecular marker 1 kb plus DNA ladder.

To confirm the specificity of the primer sets used to detect BGYMV (Table 1) and to obtain a DNA fragment of BGYMV A, we used the degenerate primers PAL1v1978 & PAR1c715 designed to detect the component A of several members of the genus *Begomovirus* [25]. Those primers direct the amplification of a ~1.4 kb fragment. As shown in Figure 1, the primers allowed the detection of the positive control BGYMV A (Figure 1A, lanes 3 and 4) but not the component B of BGYMV-MX (Figure 1A, lane 5). Also, those results confirmed that the primers set is suitable to detect and obtain a fragment of BGYMV A in extract of both, infected plant samples and infective clones. With the set of primers according to Potter et al. [22], a ~650 bp fragment was amplified in both positive (Figure 1A, lanes 12 and 14) and negative (Figure 1A, lane 13, and lanes 15–18) controls, except for the healthy plant (Figure 1A, lane 11), indicating the low specificity of the primer set. On the other hand, the use of primer sets DMexBGYMV & RMexBGYMV (Figure 1B, lanes 3 and 4), and DUnivBGYMV & RUnivBGYMV (Figure 1B, lanes 12 and 13) resulted in the expected DNA amplification (272 and 241 bp, respectively) only in the positive controls demonstrating a good specificity.

The presence of BGYMV in nine samples of bean leaf tissues collected in commercial fields in the state of Nayarit, Mexico was confirmed by sequencing the amplified fragment with the primers PAL1v1978 & PAR1c715. The sequences had an identity between 98% and 99% with the sequence of the BGYMV-MX component A (AF173555.1). The fragments amplified with the set of primers DMexBGYMV & RMexBGYMV were highlighted based on the sharpness visualized through 1% agarose electrophoresis, whereby the fragments were selected to detect BGYMV and determine compatibility with BCMV- and BCMNV-specific primers in duplex and multiplex PCR reactions.

### 3.2. Optimization of Uniplex, Duplex and Multiplex PCR for Detection of BCMV, BCMNV and BGYMV

**Figure 2:**
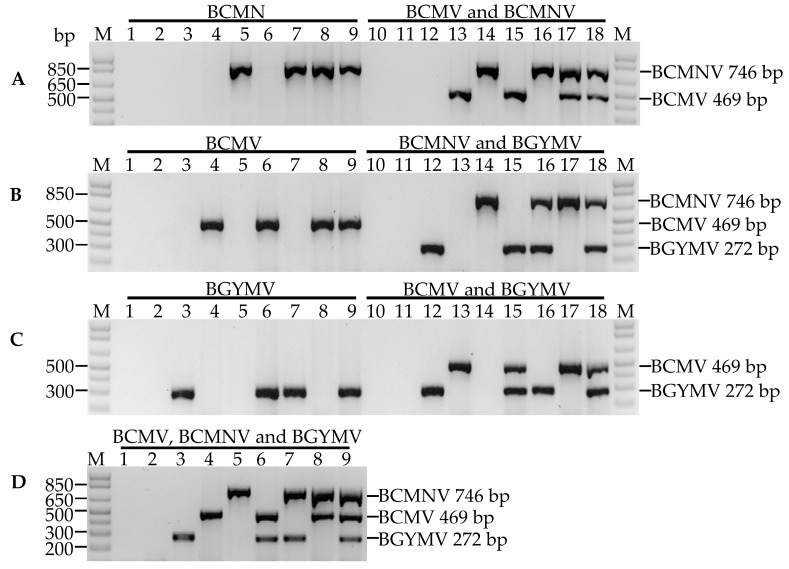
Determination of specificity and compatibility of primer pairs for the detection of bean common mosaic virus (BCMV), bean common mosaic necrotic virus (BCMNV) and bean golden yellow mosaic virus (BGYMV) in (**A**,**B**,**C**) uniplex, (**A**,**B**,**C**) duplex and (**D**) multiplex PCR. The combinations of the viruses detected with specific primers are shown above in each photograph. Lanes 1 and 10: non-template control; lanes 2 and 11: negative control (cDNA from healthy plant); lanes 3 and 12: single BGYMV control; lanes 4 and 13: single BCMV control; lanes 5 and 14: single BCMNV control; lanes 6 and 15: double BCMV and BGYMV control; lanes 7 and 16: double BCMNV and BGYMV control; lanes 8 and 17: double BCMV and BCMNV control; lanes 9 and 18: triple BCMV, BCMNV and BGYMV control. M: Molecular marker 1 kb plus DNA ladder. The sizes of the amplified product for each virus are shown at the right of the figure.

To optimize uniplex, duplex and multiplex detection we used as template in the positive control PCR reactions, the DNA from individual viruses at single, double and triple equimolar mixtures. In the uniplex detection of BCMNV, a ~746 bp fragment was amplified only in the single-, double- and triple-virus template containing the BCMNV (Figure 2A, lanes 5, 7–9). A similar result was obtained with uniplex detection of BCMV and BGYMV, in which a fragment of ~469 bp (Figure 2B, lanes 4, 6, 8 and 9) and one of ~272 bp (Figure 2C, lanes 3, 6, 7 and 9) were amplified only in the single-, double- and triple-virus templates containing the specific virus for which the primers were designed. This specificity was also observed in the duplex detection of BCMV and BCMNV (Figure 2A, lanes 13–18), BCMNV and BGYMV (Figure 2B, lanes 12, and 14–18), and BCMV and BGYMV (Figure 2C, lanes 12, 13, and 15–18), and triple (Figure 2D) where one, two or three fragments of the expected size for each primer combination (Table 1) were amplified depending on the presence of the corresponding viruses in the PCR template. These results confirmed the specificity and compatibility between primer sets to detect BGYMV, BCMV and BCMNV viruses in uniplex, duplex or multiplex reactions in single or mixed samples.

### 3.3. Composite Sampling

**Figure 3:**
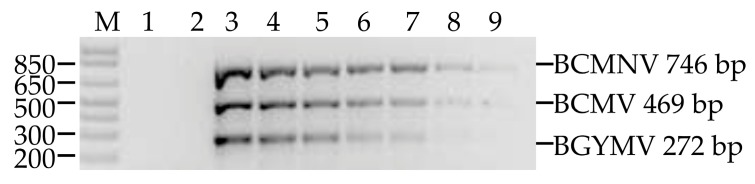
Detection of BCMV, BCMNV and BGYMV in composite samples. M: Molecular marker 1 kb plus DNA ladder. Lane 1: non-template control; lane 2: negative control (cDNA from healthy plants); lane 3: positive control (cDNA from common bean infected with BCMV, BCMNV and BGYMV); lanes 4, 5, 6, 7, 8 and 9: dilution 1:1, 1:2, 1:3, 1:4, 1:5, and 1:6, respectively.

The three viruses were detected in two-step RT-PCR tests with up to 1:4 dilutions of triple-virus infected samples diluted with cDNA of virus-free plants (Figure 3).

### 3.4. Multiplex Detection of BCMV, BCMNV and BGYMV in Samples Collected In Crop Fields

The multiplex RT-PCR was validated for applicability for detecting BCMV, BCMNV, and BGYMV in 187 potentially infected samples from different cultivars of common bean collected in rainfed and irrigated commercial bean fields from eight municipalities in Guanajuato, four in Jalisco, and 11 in Nayarit, Mexico (Table S1). All field-collected samples were tested through PCR for the presence of 26S rRNA to test that there were no PCR inhibitors in those samples where no viruses were detected. In the 187 samples, we detected single, double and triple infections of the BCMV, BCMNV and BGYMV viruses. The most frequently detected virus in simple infections was BCMV in 41/187 samples (21.9%), followed by BCMNV in 35/187 samples (18.7%) and BGYMV in 14/187 samples (7.5%). Mixed infections with BCMV and BCMNV were detected in a greater proportion of samples (13/187, 7.0%), followed by BCMV and BGYMV in 12/187 samples (6.4%), and mixed infections with the three viruses, BGYMV, BCMV and BCMNV in 10/187 samples (5.3%). The least frequent mixed infection was BCMNV and BGYMV in 4/187 samples (2.1%). In 58/187 (31.0%) samples, none of the three viruses were detected (Table 2).

The highest proportion of single infections with BGYMV and BCMNV were detected in the state of Nayarit, in 8/73 (11%) and 24/73 samples (32.9%), respectively, while the highest proportion of infections with BCMV was detected in samples collected in the state of Jalisco, in 15/38 samples (39.5%; Table 2).

With respect to the identification of mixed infections, the highest proportion of double infections by BCMV and BMCNV was detected in the state of Jalisco, in 8/38 samples (21.1%), while in the samples from Nayarit, we detected the highest proportion of infections with BCMV and BGYMV in 8/73 samples (11.0%), and of BCMNV and BGYMV in 4/73 samples (5.5%), and also of triple infections with BCMV, BCMNV and BGYMV in 8/73 samples (11.0%) (Table 2). On the contrary, the state of Guanajuato was notable for the number of samples in which none of the three viruses (Table 2) were detected (44/187, 57.9%), particularly in samples from the municipalities of Celaya (22/38, 57.9%) and Huanímaro (12/14, 87.5%; Table S1).

In general, in most types of bean cultivars BCMV, BCMNV and BGYMV were detected causing single, double or triple infections. Samples with triple infections were detected in the samples from Nayarit in yellow (Amarillo 33), pinto (Pinto Saltillo) and black seeded (Jamapa) bean cultivars. In the yellow type (Amarillo 33, and Mayacoba), black seeded (Jamapa) and Peruano types, single and double infections of BCMV, BCMNV or BGYMV were detected; while in the landraces Higuerillo and Garbancillo Zarco and cultivar Dalia (Flor de Junio type) only single infections by BCMV and BGYMV were detected (Table S1).

In samples from the state of Guanajuato, two triple infections were detected, one in each cultivar: Pinto Centauro and Pinto Saltillo. In samples of Flor de Junio type, the three viruses were detected in single infections, in the pinto type, BCMV and BCMNV were detected in single infections, and in the yellow and cranberry types BGYMV or BCMNV were also detected in single infections. The single infection of BGYMV, BCMV and BCMNV was detected in a sample of a white seeded type, of a Peruano type, of a black seeded and in a Flor de Mayo type (Table S1).

Among the samples of climbing bean types (Garbancillo Zarco and a white seeded landrace) collected in the state of Jalisco, single infections with BCMV were detected, and double infections with BCMV and BCMNV; BGYMV was only detected in samples of the Flor de Junio (cultivar Marcela) and yellow seeded types; BCMV was detected in the Peruano and climbing (Garbancillo Zarco and a white seeded landrace) types; and BCMNV was observed in a sample of the climbing type and in one cultivar of the Flor de Junio type (cultivar Marcela; Table S1). It is important to mention that the cultivars sampled belong to different races: three from the Mesoamerican pool (Mesoamerican, Jalisco and Durango) and one from the Andean pool (Nueva Granada).

## 4. Discussion

In the present study, the silica gel technique was selected because samples dried with silica gel are easy to handle, require no cooling devices, dry ice, or liquid nitrogen in the field, can be stored at cold or room temperature under desiccated conditions, and if the silica gel is saturated with moisture, it can be re-used after desiccation by heating to 120 °C for two hours or even using a frying pan [29]. Silica gel has often been used to preserve leaf tissue for DNA extraction in several PCR-based techniques [30,31,32,33], and although there are few reports for total RNA preservation, this technique has been used for the detection of the single-stranded RNA virus citrus tristeza virus [34], and the viroid citrus exocortis viroid [35].

Since BCMV and BCMNV have a single-stranded, positive sense RNA (ssRNA (+)) genome and a 3′-poly(A) terminus [36], and BGYMV has a single-stranded DNA (ssDNA) bipartite genome, referred to as components A and B [37], molecular detection of these viral species requires the extraction of both total RNA and total DNA. The method of Abarshi et al. [21] was selected because it was validated with desiccated tissue and was used for the simultaneous detection of both RNA and DNA viruses [19].

For detection of BGYMV, BCMV and BCMNV, 1 μL of the first-strand cDNA reaction was used, as cDNA synthesis is performed from total nucleic acids where viral DNA is also present. In similar studies, the simultaneous detection of DNA and RNA viruses has been performed with a mixture of both cDNA and the original total nucleic acid extraction [19], with the cDNA synthesized from the extraction of total nucleic acids in single-step [17] and two-step [18] RT-PCR reactions.

To detect BGYMV we initially used the pair of specific primers PBGYPRc122 & PBGYMPv2049 [22]. However, we observed the amplification of non-specific fragments with the components A and B of the begomoviruses BGYMV, PHYVV and PepGMV (Figure 1). In addition, the size of the amplicons was similar to that obtained with the primers used to detect BCMNV (Table 1), thus two new pairs of primers specific to component A of BGYMV were designed: DMexBGYMV & RMexBGYMV, and DUnivBGYMV & RUnivBGYMV (Table 1). With both sets of primers, a single fragment was amplified with the positive control whose sequence had 100% identity with the component A of BGYMV (AF173555.1). Considering that the fragments amplified with the set of primers DMexBGYMV and RMexBGYMV were clearly visualized using 1% agarose gels, this primer set was selected to detect BGYMV.

The optimization of multiplex PCRs can pose several difficulties, including poor sensitivity or specificity and/or preferential amplification of certain specific targets. The compatibility among the primers within the reaction mixture is of great technical importance. The overall success of specific amplification depends on the rate at which primers anneal to their target and the rate at which annealed primers are extended along the desired sequence. Factors affecting the optimal annealing rates include poorly designed primers, suboptimal buffer constituents and annealing temperature [38]. For the design of the set of primers used in our research, including those reported by Chiquito-Almanza et al. [14] (Table 1), standard parameters were considered, such as a length of 23–24 nt, 40–60% of GC content, a melting temperature of 54–57 °C, and ∆G > −9 kJ for hairpins, self-dimers and hetero-dimers. The formation of primer dimers or unspecific amplification products increased in the multiplex PCR because of the presence of more than one primer pair. The primers DMexBGYMV, RMexBGYMV, D1BCMV, D1BCMNV and RU in a concentration 0.1 M, displayed compatibility at an annealing temperature of 64 °C and amplified specific unique fragments in the samples infected with a single virus and in the double or triple mixtures of the positive controls in uniplex or multiplex reactions (Figure 2A–D), thus, there was no need to evaluate the primers at different concentrations.

An alternative to reduce detection costs, particularly when analysing a large number of samples, is to use a composite sample, whose effectiveness depends on factors such as the sensitivity of the technique, sample size, virus concentration in the infected tissue and the effect of dilution of infected samples with uninfected ones [21]. In our research, the dilution of the sample infected with the three viruses was made with cDNA from healthy plants, which is different from using pure water as diluent to simulate what occurs when a composite sample from several plants is used. The three viruses were detected all the way to the fourth dilution (1:4), which indicates that this technique allows for the identification of the three viruses in composite samples of up to five plants with only one being infected, therefore reducing the costs of detection in comparison with individual sampling.

The detection of different proportions of single and mixed BCMV, BCMNV and BGYMV infections in the samples from each of the sampled states (Table 2), together with the information of affected bean cultivars and seed types (Table S1) provides a panoramic view that contributes to define the necessary actions to reduce the damage that these viruses cause in the sampled area. Since the main form of dissemination of begomoviruses is mediated through the *Bemisia tabaci* vector [12] and BCMV and BCMNV are seed-borne [10], the highest proportion of mixed infections detected in the state of Nayarit suggests that climatic conditions and cultural practices, such as the exchange of seed between producers or the use of grain as seed, favour the presence of the vector and the dissemination of viruses. The presence of mixed infections is of particular relevance because recombination between viral species is an important source of genetic variation [39,40,41] that can promote the adaptation of the virus, modifying its virulence and/or the range of hosts [42,43].

Although in our research only the presence of BGYMV was determined, the recombination among *Begomovirus* is also a common phenomenon that drives the evolution of new species and viral strains [44,45]. In dry beans, some recombinants of BCMV and BCMNV of high virulence have been identified [46]; for example, the recombinant strain BCMNV NL-3K provokes earlier symptoms and more severe than BCMNV NL-3 [47]; the isolate BCMV RU1-OR overcomes the resistance conferred by the *bc-2^2^* allele [48]. Other BCMV recombinants, such as the isolates RU-1M [49] and 1755a [50], display interesting characteristics, the former is capable of inducing whole plant necrosis at temperatures below 30 °C in common bean plants carrying the dominant *I* gene, and the second overcomes the resistance of the *bc-3* allele, two of the most effective resistance genes used in common bean breeding programmes.

The common bean samples used in this research were collected based on the presence of presumptive viral symptoms, including general stunting, deformation of leaves, crinkled, mosaic patterns, chlorosis, leaf rolling, and leaf curling (Table S1). However, virus symptoms are often easily confused with nutrient deficiencies and herbicide injury [1,51]. Symptoms resulting from nutritional deficiency may show some similarities to mosaic or mottling symptoms reflecting virus infection [51], and herbicides can cause general stunting, leaf cupping, crinkling, strapping, yellow chlorosis, interveinal chlorosis [52] or extreme leaf deformity, which may be confused with viral diseases [1]. It is likely that the symptoms observed in 30.9% of the samples in which BCMV, BCMNV, or BGYMV were not detected were the result of infection with a different virus or to one of the aforementioned causes. However, elucidating the aetiology of symptoms observed in these samples implies a process that is beyond the scope of the present study.

## 5. Conclusions

A reliable, specific, and sensitive multiplex RT-PCR technique was developed for the detection and differentiation of BCMV, BCMNV, and BGYMV, three important viruses that infect the common bean. This technique is useful for routine molecular diagnosis and epidemiological studies of the common bean. Regardless of the type of common bean, BCMV, BCMNV and BGYMV were detected in samples from the three states. The identification of mixed infections in Guanajuato, Jalisco and Nayarit, with these three virus species, suggests the potential for the evolution of new strains of *Potyvirus* and *Begomovirus* through recombination. It is clear that there is a need for the deployment of all resistant genes available in the development of dry bean resistant cultivars for these states.

## Figures and Tables

**Table 1:** Primers used in the optimization for uniplex, duplex and multiplex detection of bean common mosaic virus (BCMV), bean common mosaic necrotic virus (BCMNV), and bean golden yellow mosaic virus (BGYMV).

Target		Primer		Location
Virus	Gene	Name	Sequence (5′–3′)	(nt)
*Begomovirus*	*AC1*	PAL1v1978 ^a^	GCATCTGCAGGCCCACATYGTCTTYCCNGT	1956–1975 ^e^
	*AV1*	PAR1c715 ^a^	GATTTCTGCAGTTDATRTTYTCRTCCATCCA	712–731 ^e^
BGYMV	*CR*	PBGYPRc122 ^b^	CGTGAGTGAATCTGATAATTCAMGAG	121–146 ^f^
	*BC1*	PBGYMPv2049 ^b^	CTGCGACTGAATCTYGCAGATARTT	2049–2073 ^f^
	*AC1*	DUnivBGYMV ^c^	GAATGATGACAACGGAAATGGAGG	2084–2107 ^e^
	*AC1*	RUnivBGYMV ^c^	CACAATCGAATGGGGACAATTCC	2302–2324 ^e^
	*AC1*	DMexBGYMV ^c^	GATGAATGATGACAACGGAAATGG	2081–2104 ^e^
	*AC1*	RMexBGYMV ^c^	TCAAGGCATACATCGACAAAGGTG	2329–2352 ^e^
BCMV	*CP*	D1BCMV ^d^	AAATGTGGTACAATGCTGTGAAGG	9267–9290 ^g^
	*CP*	RU ^d^	TCAGTATTCTCGCTGGTTGTTGC	9713–9735 ^g^
BCMNV	*CP*	D1BCMNV ^d^	GAGGTGTATGAATCCGTGTCAACA	8558–8581 ^h^
	*CP*	RU ^d^	TCAGTATTCTCGCTGGTTGTTGC	9281–9303 ^h^

^a^ Rojas et al. [25]; ^b^ Potter et al. [22]; ^c^ The present study; ^d^ Chiquito-Almanza et al. [14]. The targeting nucleotide locations are based on ^e^ BGYMV-MX strain DNA-A (GenBank accession number: AF173555); ^f^ BGYMV-MX strain DNA-B (GenBank accession number: AF173556); ^g^ BCMV NL-4 strain (GenBank accession number: DQ666332); and ^h^ BCMNV NL-3 strain (GenBank accession number: NC_004047).

**Table 2:** Detection BGYMV, BCMV, and BCMNV in common bean samples collected in rainfed commercial bean fields from Guanajuato, Jalisco, and Nayarit Mexico.

State	No. of Samples	Virus Detected							
		BGYMV	BCMV	BCMNV	BCMV/BCMNV	BCMV/BGYMV	BCMNV/BGYMV	BCMV/BCMNV/BGYMV	None Detected
Guanajuato	76	4 (5.3%)	16 (21.1%)	7 (9.2%)	–	3 (3.9%)	–	2 (2.6%)	44 (57.9%)
Jalisco	38	2 (5.3%)	15 (39.5%)	4 (10.5%)	8 (21.1%)	1 (2.6%)	–	–	8 (21.1%)
Nayarit	73	8 (11.0%)	10 (13.7%)	24 (32.9%)	5 (6.8%)	8 (11.0%)	4 (5.5%)	8 (11.0%)	6 (8.2%)
Total	187	14 (7.5%)	41 (21.9%)	35 (18.7%)	13 (7.0%)	12 (6.4%)	4 (2.1%)	10 (5.3%)	58 (31.0%)

–: None detected.

## Supplementary Materials

The following are available online at www.mdpi.com/1999-4915/9/4/63/s1, Table S1: Common beans collected samples in states of Guanajuato, Jalisco and Nayarit, Mexico.
